# ACKNOWLEDGMENTS TO REVIEWERS OF “INTERNATIONAL JOURNAL OF OCCUPATIONAL MEDICINE AND ENVIRONMENTAL HEALTH” IN 2024

**DOI:** 10.13075/ijomeh.1896.02589

**Published:** 2025

The Editors of the “International Journal of Occupational Medicine and Environmental Health” wish to thank our reviewers for giving their time and expertise to review papers between January and December 2024. The Editors would like to extend their gratitude and recognition regardless of whether the papers they reviewed were finally published.

Dayana Agudelo-Castaneda (Colombia)

Abbas Baghani (Iran)

Łukasz Balwicki (Poland)

Maciej Białorudzki (Poland)

Michał Biegała (Poland)

Bartosz Bilski (Poland)

Petri Bockerman (Finland)

Francesca Borghi (Italy)

Alicja Bortkiewicz (Poland)

Katarzyna Brukało (Poland)

David Carpenter (United States)

Maciej Chałubiński (Poland)

Marcin Cyprowski (Poland)

Joanna Czarnota-Bojarska (Poland)

Sławomir Czerczak (Poland)

Luca Di Giampaolo (Italy)

Magdalena Długosz-Lisiecka (Poland)

Anna Dzielska (Poland)

Kamila Faleńczyk (Poland)

Tommaso Filippini (Italy)

Anne Fink (United States)

Marion Freyer (Germany)

Anna Garus-Pakowska (Poland)

Klaus Golka (Germany)

Krystyna Golonka (Poland)

Javier González Caballero (Spain)

Zheng Guo (Australia)

Wojciech Hanke (Poland)

Arleta Hrehorowicz (Poland)

Zbigniew Izdebski (Poland)

Rakesh Jalali (Poland)

Magdalena Janc (Poland)

Paweł Jurek (Poland)

Mo-Yeol Kang (South Korea)

Maria Katsaouni (Greece)

Bollen Kenneth (United States)

Krzysztof Klukowski (Poland)

Katarzyna Konieczko (Poland)

Małgorzata Kowalska (Poland)

Anna Kozajda (Poland)

Alicja Kozakiewicz (Poland)

Anna Krakowiak (Poland)

Aldona Kubica (Poland)

Marcin Kurowski (Poland)

Ewa Lipiec (Poland)

Małgorzata Lipowska (Poland)

Andreas Lucero (Chile)

Aurelio Luna (Spain)

Paweł Majak (Poland)

Renata Majewska (Poland)

Teresa Makowiec-Dąbrowska (Poland)

Marta Malinowska-Cieślik (Poland)

Ginevra Malta (Italy)

Paweł Małecki (Poland)

Artur Mamcarz (Poland)

Joanna Mazur (Poland)

Gabriele Messina (Italy)

Hanns Moshammer (Austria)

Albert Nienhaus (Germany)

Mine Ocaktan (Turkey)

Leszek Ośródka (Poland)

Agnieszka Pac (Poland)

Magdalena Palacz (Poland)

Anna Paszkowska-Rogacz (Poland)

Małgorzata Pawlaczyk-Łuszczyńska (Poland)

Krystyna Pawlas (Poland)

Beata Pepłońska (Poland)

Jarosław Pinkas (Poland)

Dariusz Pleban (Poland)

Kinga Polańska (Poland)

Diana Popescu (Romania)

Krzysztof Przewoźniak (Poland)

Vladimira Puklova (Czech Republic)

Ewa Puszczałowska-Lizis (Poland)

Sylwiusz Retowski (Poland)

Anna Rogozińska-Pawełczyk (Poland)

Robert Sabiniewicz (Poland)

Petros Savourdos (Greece)

David Sipos (Hungary)

Elżbieta Sochacka-Tatara (Poland)

Eliska Sovova (Czech Republic)

Elżbieta Stawicka (Poland)

Anna Stępińska (Poland)

Agata Sudolska (Poland)

Robert Susło (Poland)

Malwina Szpitalak (Poland)

Anna Szylling (Poland)

Beata Świątkowska (Poland)

Katarzyna Talaga-Ćwiertnia (Poland)

Beatrice Thielmann (Germany)

Marco Vinceti (Italy)

Maria Widerszal-Bazyl (Poland)

Bartosz Wielgomas (Poland)

Jolanta Wierzba (Poland)

Marta Wiszniewska (Poland)

Marta Witkowska (Poland)

Małgorzata Wrzesień (Poland)

Anna Zalewska-Janowska (Poland)

Ewa Zamysłowska-Szmytke (Poland)

Dorota Zawadzka (Poland)

Xin-Yu Zhang (China)

